# Dysphagia Management in the Emergency Department: Using Concept Mapping to Identify Actionable Change to Improve Services

**DOI:** 10.1007/s00455-023-10651-5

**Published:** 2024-01-11

**Authors:** Pranika B. Lal, Elizabeth C. Ward, Laurelie R. Wishart, Jasmine Foley, Maria Schwarz, Marnie Seabrook, Carolann O’Donnell, Anne Coccetti

**Affiliations:** 1 School of Health and Rehabilitation Sciences, The University of Queensland, St Lucia, QLD Australia; 2 Speech Pathology Department, Logan Hospital, Metro South Hospital & Health Service, Meadowbrook, QLD Australia; 3 Centre for Functioning and Health Research, Metro South Hospital & Health Service, Buranda, QLD Australia; 4 Logan Hospital, Meadowbrook, QLD Australia; 5 Department of Emergency, Logan Hospital, Metro South Hospital & Health Service, Meadowbrook, QLD Australia

**Keywords:** Dysphagia, Swallowing, Emergency department, Speech-language pathology, Concept mapping

## Abstract

**Background:**

Integrated speech-language pathology (SLP) services within the emergency department (ED) may facilitate timely dysphagia management. However, there are multiple patient and logistical factors specific to the ED that challenge the delivery of optimal dysphagia referral and management practices within this setting. The aim of the current study was to engage a stakeholder group to identify prioritised, actionable goals that could help enhance dysphagia management within the ED.

**Methods and Procedures:**

Applying concept mapping methodology, 16 ED stakeholders from SLP, medical, nursing, and leadership participated in semi-structured interviews to develop action statements which were sorted and ranked for importance and changeability. Multidimensional scaling and hierarchical cluster analysis were used to organise data in clusters with unifying themes before statements were ranked by importance and changeability.

**Outcomes and Results:**

Stakeholders identified 53 unique statements, grouped into 8 clusters. Review of the 8 clusters identified 3 overarching aspects for change: (a) Improving processes related to identification and referral of patients as well as communication; (b) Teamwork and collaboration amongst the ED multidisciplinary team and SLP; and (c) Improving staffing and access to training resources for SLP and nursing teams.

Seventeen statements were within the Go-zone rated highest for importance and changeability) with the highest rated statement being: Clear documentation by SLP re: recommendations.

**Conclusion:**

The current data identified multiple aspects of service provision that require change to facilitate improved dysphagia referral and management services in the ED. Collaborative actions are required by both SLP and the ED multidisciplinary team to help optimise dysphagia services.

**Supplementary Information:**

The online version contains supplementary material available at 10.1007/s00455-023-10651-5.

## Introduction

The emergency department (ED) plays a key role within the health care system. The ED often serves as the first point of contact to provide urgent, critical care to patients presenting with life-threatening conditions, sudden illness or injury, as well as those with primary and secondary diseases [1–4]. Additionally, emergency departments coordinate patient care/flow and are often the entry point to ongoing inpatient hospital care [3].

Globally, there has been an increase in demand for ED services [5]. In Australia, 8.8 million patients presented to emergency departments during the 2020–2021 financial year [4]. This demand for ED services has been multifactorial and attributed to the ageing population, reduced access to primary care services, ease of access and convenience of the ED, and inappropriate attendances for non-urgent medical conditions [5]. Consequently, this has impacted ED services, with prolonged wait times, extended length of stay in the ED, and inpatient bed pressures leading to overcrowding in the ED, and subsequent strain on staffing and financial resources [6]. Use of strategies such as short stay units and multidisciplinary care coordination as well as inclusion of allied health services within ED has been adopted to mitigate issues related to overcrowding and support efficient patient flow [7, 8].

The complexity of the ED environment, as well as challenges created by time pressures and the short stay nature of the ED setting can impact the implementation of services. These issues have particularly been recognised as factors challenging the implementation of dysphagia management services in the ED [9–11]. Recent studies by Lal et al. [9, 10] examined factors that impact on speech-language pathology (SLP) service delivery in the ED. Findings revealed that within the Australian ED context, there is variability regarding management practices, with dysphagia management often including a combination of both nursing-led dysphagia screening as well as SLP services following referral by the ED multidisciplinary team (MDT). Further, the study findings identified that only a small number of SLP services have what was termed an “expanded” service model, which involved SLP proactively sourcing referrals from the ED population, rather than waiting for the ED team referrals [9, 10]. It was also found that the prioritisation of dysphagia management in the Australian ED setting has been primarily focussed on screening dysphagia within the stroke population, due to practice guideline requirements for this population [9].

Recognising there are challenges to the delivery of dysphagia services in the ED, Lal et al. [10] examined the barriers impacting service delivery in the ED through qualitative interviews with SLP staff. These clinicians cited patient-related factors, the ED setting, SLP service factors, and perceptions of dysphagia management as key themes which impacted service delivery. For services that utilised an expanded model, analysis using the Consolidated Framework for Implementation Research, CFIR, [12] identified that constructs of adaptability, cost, compatibility, and available resources were all barriers to implementing SLP services in the ED. Prior research into the delivery of nursing-led screening in the ED had reported comparable findings, identifying the need for time efficient assessments and noting the impact of increased patient acuity within the ED on dysphagia service provision [11].

Whilst this prior research has identified a number of barriers to dysphagia service delivery in the ED, as yet there has been no systematic study of what strategies and changes are needed to help overcome these barriers. In addition, prior work has primarily focussed on SLP and their perceptions of dysphagia service implementation in the ED. In order to improve dysphagia management for patients within the ED, more thorough understanding of the service and clinical issues from the perspective of the full ED MDT is needed, engaging SLP as well as ED medical and nursing staff. Therefore, the aim of this study was to identify prioritised actions for service change to enhance dysphagia management processes and SLP service delivery within the ED by engaging a multidisciplinary ED stakeholder group. To address this aim, a concept mapping approach was used to engage with stakeholders, including speech-language pathologists, ED MDT, and SLP and ED leadership teams. This methodology enables the generation of statements that are important and changeable to help identify issues to address for service change.

## Methods

### Study Design

The current study used concept mapping, a methodology widely used within health care contexts to plan and evaluate services as well as to understand the perspectives of stakeholders [13]. It is a mixed methods approach, integrating qualitative and quantitative approaches for data collection and analysis [14] that provides a framework for evaluation and incorporating stakeholder feedback, in order to guide change [15]. Stakeholders may present with different views including varying priorities; however, concept mapping allows for organisation of common issues to then identify prioritised areas for change [14]. The methodology involves multi-step processes which includes (1) preparation, (2) statement generation, (3) structuring, (4) representation, (5) interpretation, and (6) utilisation. The current study involved only stages 1 to 5 (as detailed in Table 1), with the outcomes of these stages intended to inform stage (6) in a future service redesign and implementation study. The research was conducted within a single health service and full ethical clearance was obtained and all participants provided informed written consent prior to participation.

**Table 1:** Concept mapping processes

Stage 1: Preparation	Semi-structured interviews conducted with participants (either as a professional group or individually) to discuss dysphagia management and challenges faced in the ED.
Stage 2: Statement Generation	Participants brainstormed statements to address issues and solutions related to dysphagia management in the ED. The collective statements from all groups were reviewed and duplicates removed to generate a final list of statements.
Stage 3: Structuring	Each participant sorted all the statements into groups based on similarity and ranked the statements according to importance and changeability.
Stage 4/5: Representation and Interpretation	Statement data were analysed using R-Cmap. Multivariate analysis produced:
	(1) Dendrogram to identify statement clusters which was reviewed by the research team to generate overarching themes for each cluster
	(2) Cluster rating map to identify relationships between each cluster
	(3) Pattern matching graph to show the average ratings for importance and changeability for each cluster
	(4) Go-zone plot to depict the average ratings for importance and changeability for each statement

### Setting and Context

This study involved SLP and ED staff from a large acute care public hospital recognised as one of the busiest in the state of Queensland, Australia [16] with more than 101,000 presentations to the ED in the 2020–2021 financial year. The ED provides services to both adult and paediatric patients within the 70-bed unit. The SLP department at this facility provides an in-reach, proactive service to patients in the ED with service provision 7 days of the week. A multi-disciplinary approach to dysphagia management is undertaken at this study site. In this model, SLP services attend the ED and conduct clinical swallowing assessments (± communication assessments as clinically indicated) on at-risk patients. In addition, the Royal Brisbane and Women’s Hospital Dysphagia Screening Tool (RBWH DST [17] is used by nursing staff with patients who present to ED outside of SLP working hours to help inform initial dysphagia management whilst in the ED. The RBWH DST process can be completed with patients who present with stroke symptoms or with other conditions if deemed to be at risk of aspiration.

To access SLP services, the facility utilises 2 referral pathways. The first involves referrals initiated by the ED MDT, based on patient’s presenting condition (such as stroke, aspiration pneumonia), as well as any observed and/or reported symptoms of dysphagia. The second pathway involves the proactive, SLP-led identification of potential patients. For the proactive SLP-led referral pathway, SLP staff monitor the ED presentation list to identify patients who may benefit from early dysphagia assessment based on presenting symptoms or conditions (e.g. neurological symptoms including slurred speech, facial weakness and unilateral weakness, intracerebral haemorrhage, exacerbation of progressive degenerative disease, aspiration pneumonia, difficulties swallowing or with secretion management, and cancer (brain, head, and neck only)). As this site uses both types of referral pathways, it is an example of an “expanded service model” as defined by [10]) and is representative of a small number of services across Australia that utilise this type of service model. It is because this site represented an expanded service model, with ED staff, nursing, and SLP services all playing a role in dysphagia identification and management in the ED that this facility was considered for this study.

### Participants

Purposive sampling was undertaken to ensure all key stakeholder groups at the facility were represented. ED medical and nursing staff, SLP clinicians, and personnel in managerial positions of both ED and allied health services were invited to participate in the study. Inclusion criteria for clinicians (SLP, medical, and nursing) included experience of working in the ED at the study site, whilst for managers, knowledge of processes within ED was required. Clinicians who had no experience of working in the ED of the study site were excluded from the study. An expression of interest (EOI) was disseminated via the ED clinical leads to medical and nursing staff as well as to the SLP department to support recruitment. No incentive was provided to participate in the study.

A total of 16 staff members across the 4 groups consented to participate in the study including 3 medical officers, 6 nurses, 4 speech-language pathologists, and 3 staff in managerial positions. The study included 75% female and 25% male participants. The years of experience working in their respective professions included a range of 6–29 years.

### Concept Mapping Procedure

#### Stage 1: Preparation

All 16 participants took part in semi-structured interviews which included a brainstorming component. Interviews were conducted in groups for nursing and SLP staff; however, for the medical and executive staff, individual interviews were organised due to challenges of the ED work environment and logistical issues due to ongoing COVID-19 restrictions during the time of data collection. Interviews were conducted by the lead study investigator either via face to face, videoconference, or teleconference depending on the preference of the participants and any COVID restrictions. The semi-structured interview guide contained open-ended questions used to stimulate discussion with participants asked to reflect on the current dysphagia management processes in the ED of the health facility and challenges experienced within this clinical setting and to identify potential solutions to the challenges they had identified.

#### Stage 2: Statement Generation

For the final task during the interviews, participants were required to brainstorm ideas that currently supported or enhanced dysphagia management as well as identify areas for improvement specifically pertaining to dysphagia management within the ED. These ideas were worded into key statements focussing on a specific topic. The list of statements generated within the session was reviewed as a group/confirmed with each individual to check for understanding and to allow for addition of any new statements if required. Once interviews with all groups/participants were completed, statements generated from all the interviews were collated. These statements were then reviewed by two investigators on the study. Any duplicate statements were omitted from the list. The final combined list included a total of 53 unique statements from the four professional groups (Refer to Supplementary Table 1).

#### Stage 3: Structuring

Using the 53 statements, participants then completed the third stage of the concept mapping process involving sorting and rating statements. Of the 16 initial participants, only 12 completed this aspect of the study. This included one medical officer, four nurses, four speech-language pathologists, and three staff in managerial positions. This attrition was due to staff leave and increased workload demands including challenges prioritising non-clinical work during the pandemic. The sorting and ranking tasks were completed by each participant independently. For the sorting task, each participant was provided with the 53 statements, on individual cards, and instructed to sort them into groups/themes/categories as they deemed appropriate. There were no restrictions regarding the number of groups or number of statements in each group. After grouping the statements, the participants secured each pile using staples or clips to ensure the statements were not mixed and returned these to the lead investigator. For the ranking task, participants were provided with the list of the collective 53 statements, and they were asked to read and then rank each statement using a 4-point scale for both “importance” and “changeability” (1-not important/changeable and 4-extremely important/easily changeable).

#### Stage 4/5: Representation and Interpretation

Data from the sorting and rating tasks was then entered into excel. The data were then analysed using R-CMap, a concept mapping software [18]. A summary of the steps involved in the data analysis and interpretation is included below within the results section to aid readability. The sequence of the multivariate analysis and interpretation involved (1) Multidimensional scaling to determine similarity and identify any relationship between the statements generated; (2) Hierarchical cluster analysis by grouping similar statements together to identify an overarching theme for each cluster; (3) Generation of a cluster rating map and pattern-matching graph illustrating the importance and changeability rating for the clusters identified in the earlier step; and (4) Generation of Go-zone scatterplot allowing for comparison of importance and changeability scores for each of the statements generated by the participants.

## Results

Both the interpretation and detailed description of the analysis process are discussed within this section. Using multidimensional scaling, a two-dimensional plot was produced to provide a visual comparison of the level of similarity between the statements (Refer to Supplementary Fig. 1). The distances between the statements indicates the level of similarity between the statements generated as judged by the participants. The more often statements were sorted into the same group (i.e. perceived to be conceptually similar), the closer the distance on the plot. For example, statement 21 “Improve [electronic medical record] functionality to prompt for RBWH DST to be completed” and statement 23 “Maximise [electronic medical record] functionality to search for [key] words to identify patients who require dysphagia assessment to minimise human error” were within close proximity on the plot suggesting that a high number of participants grouped these statements together. This step in the analysis is also used to determine the relationship between the raw data and processed data by calculating the stress value, which allows for the statistical strength/reliability of the data to be assessed. Stress values range from 0 to 1, with lower stress values preferred as they signify a better fit [19]). The reported acceptable range for concept mapping is 0.21–0.37 [14]. The stress value of the current study was 0.299, which indicates the data are reliable to proceed with the next stages of analysis.

**Fig. 1:**
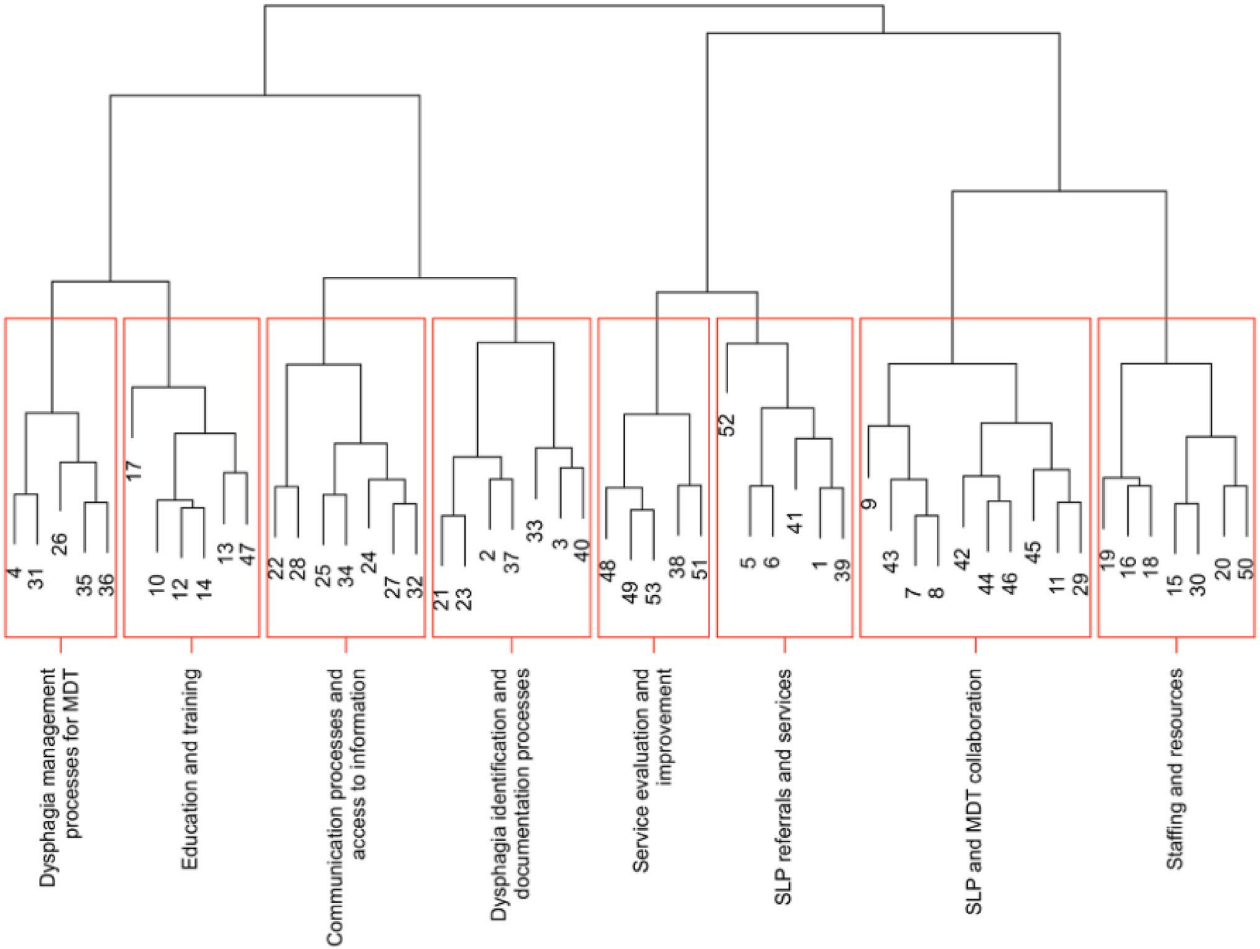
Dendrogram of meaningful cluster themes

Following multidimensional scaling, statements were then partitioned into clusters/groups based on how they were sorted by participants, using hierarchical cluster analysis. The dendrogram output displaying the clusters of statements is presented in Fig. 1. For this data, 8 discreet clusters were identified (Fig. 1). The statements within each of the 8 clusters were reviewed and the research team labelled the clusters to best represent the nature of the statements within each cluster. The labels for the 8 discreet clusters included *Dysphagia Management Processes for MDT* (5 statements); *Education and Training* (6 statements); *Communication Processes and Access to Information* (7 statements); *Dysphagia Identification and Documentation Processes* (7 statements); *Service Evaluation and Improvement* (5 statements); *SLP Referrals and Services* (6 statements); *SLP and MDT Collaboration* (10 statements); and *Staffing and Resources* (7 statements). These clusters, the description of each, and an example statement are displayed in Table 2. The highest number of statements was contained within the cluster *SLP and MDT Collaboration* which focussed largely on the relationship of the SLP department with the ED MDT, including improved presence and engagement. The cluster *Dysphagia Identification and Documentation Processes* had the second equal number of statements and broadly included use of electronic processes for improved identification of patients with dysphagia as well as improved documentation and referral processes. The cluster *Staffing and Resources* included both improved staffing resources for the SLP to improve access as well as nursing resources to meet the demands of the ED workload. *Communication Processes and Access to Information* pertained to use of electronic medical records or similar applications to communicate processes and recommendations as well as verbal communication between staff. *Education and Training* focussed on upskilling of staff and access to in-services and education for ED staff. *SLP Referrals and Services* also contained 6 statements with emphasis on scope of improvement with regards to referral information to SLP and timing of referrals and SLP-led assessment.

**Table 2:** Details of cluster themes and mean ratings

Cluster ID	Theme	Theme descriptions	Example statement	Number of statements	Mean importance rating	Mean changeability rating
1	Dysphagia management processes for MDT	Covered aspects of clinical pathways and processes to ensure dysphagia management was prioritised by ED MDT	Streamlined dysphagia clinical pathways including care plans for communication	5	2.88	2.60
2	Education and training	Focussed on opportunities for education for ED MDT and increased awareness of services/processes	Regular in-services to nursing staff/assistants in nursing and ED staff re: RBWH DST and periodic in-services for general dysphagia management	6	2.86	2.71
3	Communication processes and access to information	Covered elements related to verbal handover as well as use of technology to communicate information relating to dysphagia management plans	Quick/easy access to information within the patient’s chart re: diet and fluids instead of having to delve through the chart to find key information	7	3.13	2.55
4	Dysphagia \identification and documentation processes	Related to improved identification and referral processes including through use of current electronic medical charts	Improved identification of patients with pre-existing dysphagia (ED MDT) to allow for provision of early dysphagia management	7	3.11	2.60
5	Service evaluation and improvement	Covered aspects of review/auditing of current service provision	Engage in research and complete audits to demonstrate need for SLP	5	2.95	2.38
6	SLP referrals and services	Discussed variability and timeliness of referrals, enhanced SLP autonomy, and improved access to SLP services	Referrals are currently “all over the shop”. Need to have consistency regarding when and how referrals are received	6	3.00	2.60
7	SLP and MDT collaboration	Discussed cultivating stronger relationships between SLP and ED MDT	There needs to be better engagement with allied health in ED to improve referral process and communication	10	2.72	2.64
8	Staffing and resources	Supported increase in staffing for SLP and nursing in ED to support care for patients with dysphagia in ED	High workforce demands of the ED setting impact on whether dysphagia management is prioritised	7	3.00	2.10

**Fig. 2:**
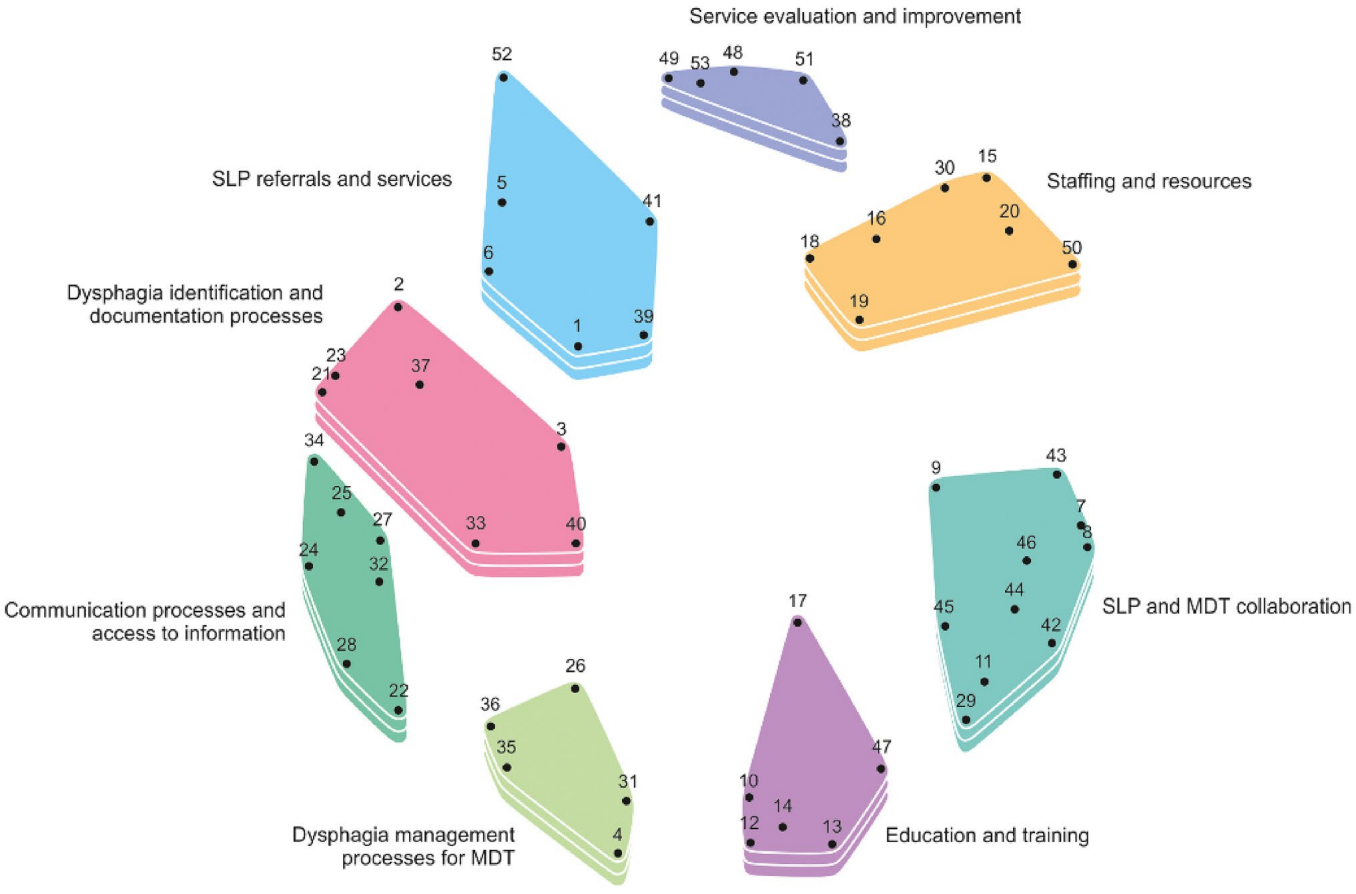
Cluster rating map for each cluster theme (by importance)

Data regarding the importance and changeability ratings were represented using 3 methods: cluster rating map, pattern matching, and Go-zone data. The cluster rating map depicts the relationship of statements/clusters to each other, illustrated by how closely adjacent the clusters are on the map (Fig. 2). For example, statements within the cluster *Education and training* was considered least related to *Service Evaluation and Improvement* and therefore located further apart on the map. In contrast, the cluster *Dysphagia Identification and Documentation Processes* and *Communication Processes and Access to Information* were in closer proximity indicating that the statements in these clusters were more likely to be related. Then, the level of importance of a cluster is reflected by the number of layers in each stack, with more layers equivalent to a high average importance rating for the statements. The cluster *Communication Processes and Access to Information* was most highly rated on importance with an average of 3.13. The size of the clusters reflects the level of coherence/agreeance (i.e. how frequently the participants sorted the statements into the same piles). Clusters of statements with higher agreement are graphically represented by clusters that are smaller in size. Finally, the distance between each cluster boundary suggest the level of distinctiveness of each theme.

**Fig. 3:**
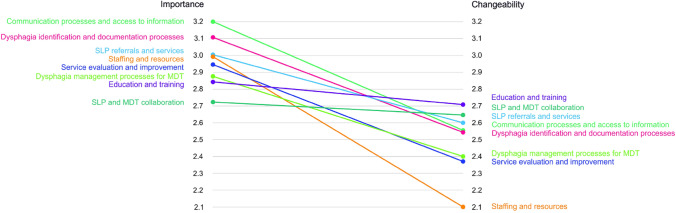
Pattern matching comparing importance and changeability of clusters

The next stage of analysis involved generation of a pattern-matching graph (Fig. 3), which depicts the bivariant relationship between importance and changeability across the clusters identified [13]). The average rating of each of the statements within the clusters is plotted on a graph along the vertical axis with the gradient line representing where a positive or a negative relationship exists. Using this information, general concepts relating to dysphagia management services within the ED can be identified for future goal setting. The relationship between importance and changeability was negative for all clusters in this study indicating that participants rated most of the statements as important but also identified that these would be challenging to change/implement. Five clusters (*Communication Processes and Access to Information*, *Dysphagia Identification and Documentation Processes*, *Staffing and Resources*, *SLP Referrals and Services,* and *Service Evaluation and Improvement*) had a rating of 3 or higher. However, the importance ratings for these clusters did not always translate to changeability ratings. There were four clusters with changeability ratings of 2.5 or more. The clusters *Education and Training* and *SLP and MDT Collaboration* were deemed to be most changeable. Only two clusters with high importance ratings were also rated as most changeable: *SLP Referrals and Service*s and *Communication Processes and Access to Information*. However, two of the clusters with the highest importance ratings (*Staffing and Resources* and *Service Evaluation and Improvement*) were considered most difficult to change.

**Fig. 4:**
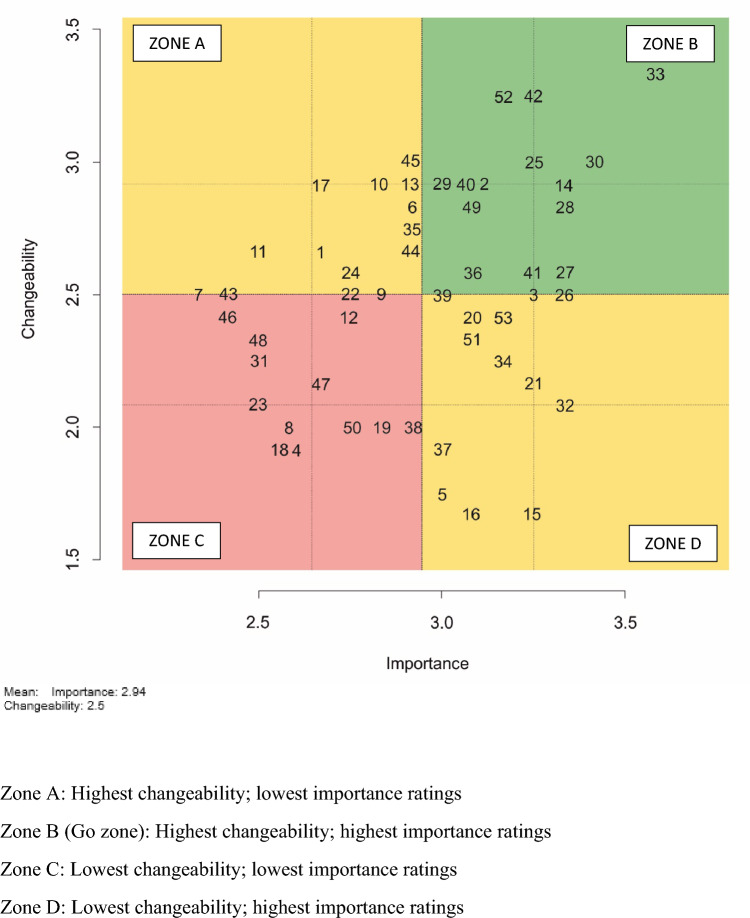
Go-zone plot illustrating the plot of all statements across importance and changeability. The position of the statements is determined by the average ratings for importance and changeability. Zone A: Highest changeability; lowest importance ratings; Zone B (Go-zone): Highest changeability; highest importance ratings; Zone C: Lowest changeability; lowest importance ratings; Zone D: Lowest changeability; highest importance ratings

The final analysis stage involved a graphic representation of importance ratings in comparison to changeability ratings for each individual statement, using a bivariant plot (referred to as the Go-zone plot). In contrast to the cluster rating maps which focusses on the clusters, the focus of the Go-zone plot is on the individual statements and can therefore be used to guide development of specific goals [13]. The Go-zone plot is divided into quadrants with y-axis (changeability) and x-axis (importance) depicting the average values for each of the 53 statements (Fig. 4). The statements with highest importance as well as changeability scores were plotted in the “Go-zone” (Zone B) thereby guiding high-priority actionable goals. Contrastingly, statements with lowest scores on both variables fall in Zone C suggesting consensus of limited perceived relevance and ability to be changed. The left upper quadrant represented statements with the higher changeability scores but lower importance scores. The inverse of these rating scores was depicted in the right lower quadrant.

A total of 17 statements fell within Zone B “Go-zone” and 12 statements in Zone C (Fig. 4). The 17 individual statements that were included within the Go-zone are detailed in Table 3. As outlined in Table 3, there were statements from each of the 8 clusters contained in the Go-zone, with the largest numbers of statements in the Go-zone (*n* = 7) relating to communication issues (from the clusters *Communication Processes and Access to Information*, *Dysphagia Identification and Documentation Processes, Dysphagia Management Processes for MDT, SLP and MDT Collaboration).* A further four of the Go-zone statements related to improving early identification of dysphagia and the timing of assessment (from the clusters *Dysphagia Identification and Documentation Processes* and *SLP Referrals and Services*) (Table 3). Statement number 33 “*Clear Documentation by SLP re: Recommendations for Ease of Access”* in the Go-zone was found to have the highest rating for both importance and changeability (Fig. 4).

**Table 3:** Go-zone statements from the Go-zone plot

Statement ID	Statement	Cluster number and theme
26	Streamlining of information between SLP and MDT for getting referrals, handover, and discharge planning	Cluster 1: Dysphagia management processes for MDT
36	Streamlined dysphagia clinical pathways including care plans for communication	
14	Education of staff re: referral processes to SLP	Cluster 2: Education and training
25	Better bedside visual cues/Clear indication that the patient is on modified diet/fluids especially because ED is a busy environment (e.g. Improve clarity of bedside information re: diet to maximise patient safety)	Cluster 3: Communication processes and access to information
27	Improved communication re: premorbid diet and fluids	
28	Improved communication with kitchen to ensure patient is provided the correct diet including use of software [such as Trendcare] to communicate diet/fluid information with kitchen	
2	SLP [ED presentation] screening processes can be expanded to include early identification of high-risk clinical populations	Cluster 4: Dysphagia Identification and documentation processes
3	Improved identification of patients with pre-existing dysphagia (ED MDT) to allow for provision of early dysphagia management	
33	Clear documentation by SLP re: recommendations for ease of access	
40	There needs to be a defined process for access/referral to SLP in ED	
49	Completion of patient interviews of their experience with SLP in ED	Cluster 5: Service evaluation and improvement
39	Improve timeliness to SLP assessment for patients in ED	Cluster 6: SLP referrals and services
41	Improve accessibility to SLP services	
52	Demonstrate risk to patients by completion of clinical risk reporting when errors or adverse outcomes occur	
29	Maintaining team communication with nursing staff including advising when SLP will be there to assess patients and giving verbal feedback post assessment	Cluster 7: SLP and MDT collaboration
42	ED staff to become familiar with SLP staff to improve relationships and referrals	
30	Better access to modified diet and fluids available in the ED especially out of hours	Cluster 8: Staffing and resources

## Discussion

This study utilised concept mapping with clinicians and managers from both the ED MDT and SLP services. Use of this novel methodology allowed the identification of the top themes impacting services (i.e. the clusters) and at the statement level, allowed for identification of those statements deemed most important and changeable for improving dysphagia management within the ED of a single clinical setting. Given the opportunity to identify actions for change, participants generated a total of 53 statements focussing on a range of issues. Considering the challenges of the complex clinical ED environment, it is not surprising that these statements not only related to specific changes to the dysphagia management processes itself but also addressed challenges of service provision and the delivery of clinical care working within the busy ED setting. This study data provides direction for further development and enhancement of dysphagia management practices in the ED.

Whilst prior work has identified that there are multiple challenges with the delivery of dysphagia services within the ED [10], the current data build on that knowledge by providing a set of actionable statements that key stakeholders perceived were needed to improve their current services. These 53 unique statements were grouped by participants into eight clusters of related concepts (Table 2). Whilst each cluster of statements represented a specific aspect of SLP and ED MDT services that required change, overall across the 8 clusters there were three main aspects of ED/SLP services that were the main focus of change: (a) clusters 1, 3, 4, and 6 all contained statements about improving “processes”, including the identification of patients, referral to SLP, and improvements to written and verbal communication processes, (b) clusters 5 and 7 contained statements which focussed on the importance of “teamwork and collaboration” between SLP, medical, and nursing staff in the ED, while (c) clusters 2 and 8 contained issues related to “resources”, including improved SLP and nursing staffing as well as opportunities for training and education (Table 2). These findings highlight that improving dysphagia services will be complex, requiring multiple different strategies and involving the collaborative efforts of both the ED MDT and SLP staff, in order to achieve change.

A key feature of the concept mapping process is that each statement is considered both from the perspective of its perceived importance, but also its capacity to be enacted upon/changed. Analysis of these two dimensions revealed that all clusters were identified as more important than changeable by the participants (as represented by the negative gradient in Fig. 3). This finding has been observed in other studies that have used concept mapping to identify changes to services and reflects the awareness of the participants about the challenges of implementing service changes within a complex healthcare environment [15, 20]. However, despite there being a perception that most things were more important than changeable, 5 out of the 8 clusters received a changeability score of 2.5 or higher. This result indicates that a number of key actions were perceived as feasible and more easily undertaken to improve the state of dysphagia services within the ED. The 5 clusters identified as most changeable related to *Education and Training, SLP and MDT collaboration, SLP Referrals and Services, Communication Processes and Access to Information;* and *Dysphagia Identification and Documentation Processes*. Of these *Education and Training* was seen as the most highly changeable. Previously, Lal et al. [10] identified that SLPs considered training and education as facilitatory factors in the implementation of dysphagia services in ED; however, they also noted challenges delivering training in the ED due to high competing clinical priorities, the shift-work nature of the 24-h ED service, and high staff turnover. As such, even though participants in the current study felt training/education was something that could most easily be addressed, finding ways to deliver and sustain staff education in the ED will require novel solutions, such as the use of technology (e.g. online training) to provide flexible training delivery options and/or collaborating with the ED education team to provide inter-professional (SLP and ED staff) joint training to enhance the efficiency of training opportunities offered within the busy ED clinical caseload [21].

Despite being rated highly for importance, it was not surprising that *Staffing and Resources* was identified as the least changeable cluster, as participants recognise the impact of financial constraints at an organisational level. Within this cluster, the statements related to staffing for funding to improve accessibility to SLP services as well as nursing resources to support patients with dysphagia (e.g. with feeding assistance). Whilst there is no research on the impact of reduced nursing resources specifically on dysphagia management, it is recognised that poor staffing for both medical and nursing staff has flow on clinical and service impacts in the ED including on patient care, wait times, and patient flow and work-related stress [22, 23]. With regards to resourcing for SLP, inadequate SLP staffing would impact on responsiveness to referrals and time to assessment. Whilst ED and SLP staff perceived this to be the least changeable area, further consideration by leadership may be warranted given the known impact of inadequate staffing and resourcing on clinical workload management as well as patient care.

Analysis of the individual statements by those highly rated for both importance and changeability revealed a set of 17 statements (i.e. statements in the Go-zone, Fig. 4). Included in this group were 7 statements relating to communication. Whilst most of these were from the cluster *Communication Processes and Information,* there were statements relating to communication from 3 other clusters. These statements typically focussed on improving communication relating to diet/fluids and the communication between ED MDT and SLP staff. It is not surprising that staff value the importance of clear communication between the MDT members (and with patients) to enhance patient safety. The challenges to effective communication and team collaboration in the ED environment are recognised and include (but not limited to) high and demanding workload, multiple handovers between individuals and teams (including in-reach teams), variability in staffing, incomplete or inadequate patient histories, and presentation of patients with high-risk clinical conditions [24]. Effective and accurate communication about dysphagia management plans is particularly important for patient safety, as miscommunication can lead to patients receiving incorrect oral intake, placing them at increased risk of adverse clinical outcomes [25]. Strategies to improve communication may include SLP consultation with medical and nursing staff prior to and post-assessing a patient, timely documentation of recommendations into the patient’s medical record, consistency in use of terminology and education relating to this, and use of handover tools to structure communication [24–26].

There were also 4 statements in the Go-zone that related to improving early identification of dysphagia and the timing of assessment, which were statements from the clusters *Dysphagia Identification and Documentation Processes* and *SLP Referrals and Services.* These statements can be considered together, as the underpinning message relates to clinical and service responsiveness. Efficient identification of dysphagia risk will in turn support timely referrals and therefore assessments. One approach to support more efficient timing of dysphagia assessment could be the use of proactive SLP screening of ED presentations, rather than waiting for ED referral. As reported in the recent study by Lal et al. [27] significantly more stroke patients were identified via a SLP-initiated referral pathway and were assessed by SLP within 8 h of presentation to ED, compared to the more traditional ED-initiated referral pathway. Although the SLP department involved in the current study already engages in proactive screening of ED presentations, certain populations (e.g. post-stroke) are more easily identified at admission as needing dysphagia screening. Further development and refinement of criteria used to screen and identify potential at-risk admissions to the ED could help the early identification and management of other key clinical populations and enhance the timeliness of SLP assessment of at-risk patients within the ED.

Although the use of a SLP-initiated referral pathway may be one strategy to enhance the efficient identification of at-risk patients, the role that the ED MDT play in early dysphagia identification is also critical to optimise. ED medical and nursing staff are the first point of contact for all patients who present to ED and therefore strategies to support the ED MDT to recognise patients at risk of dysphagia on admission is also needed. This could include provision of education to ED staff on how to identify dysphagia risk and how to refer at-risk patients to SLP services. Nursing-led dysphagia screening, which is part of many ED services in Australia for the stroke population [9] could also be expanded to a broader range of clinical conditions (i.e. beyond the stroke population). Given the clinical demand of the ED caseload, strategies to streamline referrals to SLP may also assist ED staff to initiate referrals more efficiently.

Each of the statements in the Go-zone identified actions for either the SLP, ED MDT staff, or both to address. For example, the highest rated statement in the Go-zone was “clear documentation by SLP re: recommendations for ease of access”. This statement can be directly addressed by the SLP department with consideration of processes that enable consistency and clarity of documentation especially with regards to SLP recommendations. There were a further 11 Go-zone statements that will need to be addressed collaboratively by the ED MDT and SLP department, for example, streamlining and enhancing communication amongst staff, streamlining clinical pathways and processes, and developing relationships with the SLP department. Whilst there were no statements in the Go-zone that specifically identified actions required by the executive team, active engagement with leadership is recognised as central to implementing service change [28].

### Limitations

It is acknowledged that this study was an evaluation of the dysphagia services being offered within a single hospital site and within the Australian setting only and therefore, it would be expected that there will be different factors staff perceive as impacting dysphagia services within the ED at other clinical sites and in other countries. In addition, participants’ level of knowledge and experience of dysphagia management within the ED must be accounted for. Participant bias is considered as individual perceptions of importance of dysphagia within the ED service delivery model may have been influenced by the level of personal connection to the service model. This study was also completed during the peak of the COVID-19 pandemic and the resource intensive nature of the study design during this time contributed to the attrition of participants, with fewer participants completing the rating/sorting component of the study. Further, due to the high clinical demands of the ED caseload, interviews with medical officers and executive staff could not be completed with their respective groups and were instead completed individually. Therefore, medical officers and managers did not have the opportunity to brainstorm ideas as a collective professional group. It is also acknowledged that the focus of the current analysis was on those statements which fell within the Go-zone (i.e. identified as most highly important and changeable). It could be argued that prioritising only those items deemed as most important could be another way to identify issues to be prioritised in the future service change. Additionally, this study only considered the views of staff working in the ED and not of consumers. Future research should consider what consumers also deem necessary for change to dysphagia management in the ED. Finally, the scope of this study did not allow for exploration of Phase 6 of the concept mapping process, i.e. ‘utilisation’. Future studies that evaluate the impact of implementing the strategies identified in the current concept mapping process will help to guide the optimisation of dysphagia services within the ED.

## Conclusion

Concept mapping was utilised in this study to support ED MDT, SLP, and leadership teams to identify the main themes/issues impacting services and then to generate a prioritised list of actions for change to help improve dysphagia services within the ED. Key statements identified for change included improving communication between staff including in relation to diet/fluids, improving dysphagia identification for high-risk clinical populations including patients with pre-existing dysphagia, and improving accessibility to SLP services in ED. Further engagement involving ED MDT and SLP with leadership is required to integrate this data and implement processes for change.

### Supplementary Information

Below is the link to the electronic supplementary material.

Supplementary file1 (DOCX 36 KB)
