# Supplementary material for: Dysphagia Management in the Emergency Department: Using Concept Mapping to Identify Actionable Change to Improve Services
Source: Dysphagia. 2024 Jan 11;39(4):705–17. doi: 10.1007/s00455-023-10651-5 (PMC11239603; doi:10.1007/s00455-023-10651-5)
Supplement: Supplementary file 1 — Supplementary file1 (DOCX 36 KB) [file 455_2023_10651_MOESM1_ESM.docx]

Supplementary Table 1: Statements generated by participants

| No. | **Statement** |
| --- | --- |
| 1 | Referrals are currently "all over the shop'. Need to have consistency regarding when and how referrals are received. |
| 2 | SLP [ED presentation] screening processes can be expanded to include early identification of high risk clinical populations. |
| 3 | Improved identification of patients with pre-existing dysphagia to allow for provision of early dysphagia management. |
| 4 | Dysphagia management should be core practice as part of ED MDT. |
| 5 | SLP proactive screening is currently responsive, but SLP require more timely information regarding medical acuity and diagnosis. |
| 6 | SLP to have autonomy to self-identify and screen patients presenting from nursing homes (to support discharge) |
| 7 | Visual SLP identity within the ED team -other teams are easily identifiable via uniforms etc however SLP is not. |
| 8 | SLP to be recognised as part of the ED MDT including being co-located within the ED. |
| 9 | There needs to be better engagement with allied health in ED to improve referral processes and communication. |
| 10 | Regular in-services to nursing staff/assistants in nursing (AINs) and ED staff re: dysphagia screening tool (DST) and periodic in-services for general dysphagia management. |
| 11 | SLP to be present and actively contribute to medical Grand Rounds to increase understanding of the role of SLP. |
| 12 | More education (including DST training) for medical staff – registrar teaching as well as education for senior medical officers. |
| 13 | SLP information to be included as part of orientation for all staff in ED especially medical and NS. |
| 14 | Education of staff re: referral processes to SLP. |
| 15 | Better staffing resources for assistants in nursing (AINs) to support with feeding patients with dysphagia. |
| 16 | Dedicated funding for SLP in ED to improve access to SLP services. |
| 17 | Upskilling of SLP staff in ED. |
| 18 | Increased/extended hours to improve accessibility to SLP services. |
| 19 | SLP to demonstrate need for SLP in ED (this could include re-directing resources to demonstrate need for SLP services). |
| 20 | Provision of resources to assist with feeding of patients with dysphagia. |
| 21 | Improve functionality [of electronic medical record systems] to prompt for DST to be completed. |
| 22 | Better use of technology (e.g use of QR codes) to easily access work instructions and management pathways (instead of overwhelming the ED walls with information). |
| 23 | Maximise [electronic medical record] functionality to search for buzz words to identify patients who require dysphagia assessment (to minimise human error). |
| 24 | Education on use of [electronic medical record] functionality e.g. to search for diets/fluids. |
| 25 | Better bedside visual cues/clear indication that the patient is on modified diet/fluids especially because ED is a busy environment (e.g. improve clarity of bedside information re: diet to maximise patient safety). |
| 26 | Streamlining of information between SLP and MDT for getting referrals, handover, discharge planning. |
| 27 | Improved communication re: premorbid diet and fluids. |
| 28 | Improved communication with kitchen to ensure patient is provided the correct diet including use of software [such as Trendcare] to communicate diet/fluid information. |
| 29 | Maintaining team communication with nursing staff including advising when SLP will be there to assess patients and giving verbal feedback post assessment. |
| 30 | Constant reminder processes for ED medical team to think about dysphagia. |
| 31 | Quick/easy access to information within the patient chart re: diet and fluids instead of having to delve through the chart to find key information. Currently takes time to find the diet/fluids. |
| 32 | Clear documentation by SLP re: recommendations for ease of access |
| 33 | Use of alerts/risks [within electronic medical record]to improve communication re: diet and fluids |
| 34 | Use of flow charts for dysphagia management to provide regular visual reminders (e.g. what should be done before and after oral intake). |
| 35 | Streamlined dysphagia clinical pathways including care plans for efficient communication. |
| 36 | Improve timeliness of access to critical information regarding patient care to support SLP screening of ED presentations. |
| 37 | Reduced work hours/weekend hours of SLP cover impact on service provision to ED. |
| 38 | Improve timeliness to SLP assessment for patients in ED. |
| 39 | There needs to be a defined process for access/referral to SLP in ED. |
| 40 | Improve accessibility to SLP services. |
| 41 | ED staff to become familiar with SLP staff to improve relationships and referrals. |
| 42 | To have the same/consistent one SLP servicing the ED (to build familiarity) |
| 43 | SLP to attend ED MDT meetings to increase presence within ED. |
| 44 | SLP to introduce themselves to the ED team and share information about the current service. |
| 45 | SLP department to have better engagement with leadership team. |
| 46 | Leadership team to improve their awareness of the role of SLP. |
| 47 | Review of dashboard data of SLP service provision in ED and discussion at executive level. |
| 48 | Completion of patient interviews of their experience with SLP in ED. |
| 49 | High workforce demands of the ED setting impact on whether dysphagia management is prioritised. |
| 50 | Review of activity-based funding implications of having SLP in ED. |
| 51 | Demonstrate risk to patients by completion of clinical risk reporting when errors or adverse outcomes occur. |
| 52 | Engage in research and complete audits to demonstrate need for SLP. |
| 53 | Better access to modified diet and fluids available in the ED especially out of hours. |
